# Tri6 Is a Global Transcription Regulator in the Phytopathogen *Fusarium graminearum*

**DOI:** 10.1371/journal.ppat.1002266

**Published:** 2011-09-29

**Authors:** Charles G. Nasmith, Sean Walkowiak, Li Wang, Winnie W. Y. Leung, Yunchen Gong, Anne Johnston, Linda J. Harris, David S. Guttman, Rajagopal Subramaniam

**Affiliations:** 1 Agriculture and Agri-Food Canada, Ottawa, Ontario, Canada; 2 CAGEF, University of Toronto, Toronto, Ontario, Canada; Virginia Polytechnic Institute and State University, United States of America

## Abstract

In *F. graminearum*, the transcriptional regulator *Tri6* is encoded within the trichothecene gene cluster and regulates genes involved in the biosynthesis of the secondary metabolite deoxynivalenol (DON). The Tri6 protein with its Cys_2_His_2_ zinc-finger may also conform to the class of global transcription regulators. This class of global transcriptional regulators mediate various environmental cues and generally responds to the demands of cellular metabolism. To address this issue directly, we sought to find gene targets of Tri6 in *F. graminearum* grown in optimal nutrient conditions. Chromatin immunoprecipitation followed by Illumina sequencing (ChIP-Seq) revealed that in addition to identifying six genes within the trichothecene gene cluster, *Tri1, Tri3, Tri6, Tri7, Tri12* and *Tri14*, the ChIP-Seq also identified 192 additional targets potentially regulated by Tri6. Functional classification revealed that, among the annotated genes, ∼40% are associated with cellular metabolism and transport and the rest of the target genes fall into the category of signal transduction and gene expression regulation. ChIP-Seq data also revealed Tri6 has the highest affinity toward its own promoter, suggesting that this gene could be subject to self-regulation. Electro mobility shift assays (EMSA) performed on the promoter of *Tri6* with purified Tri6 protein identified a minimum binding motif of GTGA repeats as a consensus sequence. Finally, expression profiling of *F. graminearum* grown under nitrogen-limiting conditions revealed that 49 out of 198 target genes are differentially regulated by *Tri6*. The identification of potential new targets together with deciphering novel binding sites for Tri6, casts new light into the role of this transcriptional regulator in the overall growth and development of *F. graminearum*.

## Author Summary

Our knowledge of mechanisms involved in the activation and biosynthesis of DON comes largely from *in vitro* culture studies. Cumulated knowledge suggests that the physiological status of the fungus and the availability of nutrients are the main determining factors for DON production. Integration of various environmental cues to coordinate expression of secondary metabolic genes is thought to be mediated by a combination of global and pathway-specific transcription factors. While the global transcriptional factors respond to broad range of environmental cues such as the availability of carbon and nitrogen, the pathway-specific transcriptional factors regulate genes within a gene cluster. In *F. graminearum*, the transcriptional regulator Tri6 is encoded within the trichothecene gene cluster and regulates genes involved in the synthesis and transport of DON. In this report, we utilized ChIP-Seq to demonstrate that Tri6 can potentially bind to promoters and regulate genes not involved in the synthesis of DON and furthermore, many of these non-trichothecene genes are involved in various aspects of cellular metabolism, including transport and energy. Expression profiling revealed that many of the target genes are differentially regulated by Tri6, thus validating our hypothesis that Tri6 is a global regulator involved in cellular metabolism.

## Introduction

*Fusarium graminearum* Schwabe [telemorph *Gibberella zeae* (Schwein.) Petch] is the causal agent of Fusarium head blight (FHB), one of the most destructive crop diseases in temperate climes throughout the world. In addition to yield reduction, FHB is often associated with the accumulation of the secondary metabolite DON in grain [1]. DON belongs to the trichothecene family of secondary metabolites; it binds to the peptidyltransferase of ribosomes thereby inhibiting protein synthesis [2]. DON accumulates in infected plant tissues, is phytotoxic, and poses considerable health risk to consumers [3]. Although considerable effort has been expended to both detect and regulate the amount of this mycotoxin in the infected cereals, there is less information with regard to the regulation of DON biosynthesis.

Our knowledge of mechanisms involved in the biosynthesis of DON and other secondary metabolites comes largely from *in vitro* culture studies. A considerable amount of evidence gathered over many years suggested that the physiological status of the fungus and the availability of nutrients are the main contributors for secondary metabolite production [4], [5]. Other growth conditions such as pH have also been shown to influence the production of secondary metabolite in numerous fungi including *F. graminearum* [6], [7]. Recent studies have assessed the role of various carbon and nitrogen sources in the induction of DON [5], [8]. For example, while the products of the polyamine biosynthesis pathway such as agmatine and putrescine strongly influenced DON biosynthesis, the study also revealed negative effects of other nitrogenous compounds on the induction of DON [5]. Other studies have also demonstrated the importance of carbon sources in the regulation of DON biosynthesis [8]. Growth conditions modified by the addition of salt solutions, hydrogen peroxide, and various phytochemicals and fungicides have also been shown to influence DON production [9].

In addition to the physiological conditions, factors affecting fungal developmental also impact the synthesis of secondary metabolites [4]. In *Aspergillus* species, the canonical heterotrimeric G protein/cyclic AMP/protein kinase A signalling pathway involved in diverse cellular responses including cell division, morphogenesis and pathogenic development affect the production of the secondary metabolites penicillin and sterigmatocystin. For example, mutations in *fadA*, a gene encoding for the Gα subunit of the heterotrimeric G protein, negatively impacts aflatoxin biosynthesis [10], [11]. Conversely, a dominant active *fadA* mutant inhibited expression of the transcription factor AflR, resulting in the blockage of sterigmatocystin synthesis. Interestingly, introduction of the same dominant active *fadA* mutant in *F. sporotrichioides* resulted in elevated levels of T-2 toxin, suggesting conservation of signalling pathways between filamentous fungi in the regulation of secondary metabolite synthesis [12].

This level of complexity, integrating various environmental inputs to fungal development leading to the activation of secondary metabolic gene clusters, has led to the current working model which proposes a multilevel regulation of secondary metabolism by both global and pathway-specific transcription factors [6]. In this scenario, the global transcription factors would respond and integrate disparate environmental cues such as temperature, pH and various carbon and nitrogen sources. Examples include A*reA* which mediates nitrogen catabolite repression in *Aspergillus*, and PacC which mediates pH regulation of the penicillin and trichothecene gene clusters in *Aspergillus*,and *F*. *graminearum*, respectively [13], [14]. One of the characteristic features of the global regulators is that they possess Cys_2_His_2_ zinc-finger domains, important for DNA binding and regulating gene expression [6]. The pathway-specific regulators on the other hand have a characteristic Zn(II)_2_Cys_6_ zinc binuclear cluster and positively regulate expression of a specific gene cluster. This is best exemplified by AflR, which regulates the sterigmatocystin biosynthetic gene cluster in *Aspergillus*, [15]. In *F. graminearum* and *F. sporotrichioides*, the transcriptional regulator *Tri6* is encoded within the trichothecene gene cluster and regulates expression of structural genes involved in the synthesis of DON and T-2 toxin, respectively [16]–[18]. Targeted disruption of *Tri6* in both *Fusarium* species established its role as a positive regulator of trichothecene genes [16], [18], [19]. Although this criterion designated Tri6 as a pathway-specific transcriptional regulator, evidence accumulated over the past few years have suggested that Tri6 may be representative of a global transcription factor whose expression is influenced by a variety of environmental factors. In addition to possessing Cys_2_His_2_ zinc-finger domains, *Tri6* is influenced by the pH of the growth media and by a large range of nitrogen and carbon compounds [5], [8]. For example, polyamines such as agmatine and putrescine induced novel genes regulated by *Tri6* [20]. Additionally, expression profiling of FHB-infected tissues identified more than 200 genes that are not part of the trichothecene gene cluster differentially regulated by *Tri6* [16]. This included genes in the isoprenoid biosynthesis pathway which produces farnesyl pyrophosphate, an immediate precursor for trichothecenes, and genes involved in transport and virulence [16]. These observations suggested that genes outside of the trichothecene gene cluster are subject to *Tri6* regulation.

To investigate the possibility that Tri6 is a global transcriptional regulator responsive to both environmental and developmental cues, a genome wide ChIP-Seq experiment was undertaken to identify potential new targets of Tri6. Therefore, ChIP-Seq was performed with *Fusarium* grown in nutrient-rich conditions, a condition optimal to detect targets involved in both growth and development. We identified 198 potential new targets of Tri6 and functional categorization associated them with energy, metabolism and other cellular processes. Expression profiling of *Fusarium* grown in nitrogen-deprived conditions, a condition optimal for the production of trichothecenes showed that 47 of 198 targets were differentially regulated in the *tri6*Δ strain. The over expression of *Tri6* in the *tri6*Δ strain confirmed that Tri6 can auto-regulate its own expression under nutrient rich conditions. Detailed analysis of the *Tri6* promoter revealed a new tandem GTGA DNA binding site, located adjacent to the previously described binding site for Tri6. This finding, together with the identification of new targets, signifies a broader regulatory role for Tri6.

## Results

### Targets of Tri6 include structural and regulatory genes involved in metabolism

**Figure 1:**
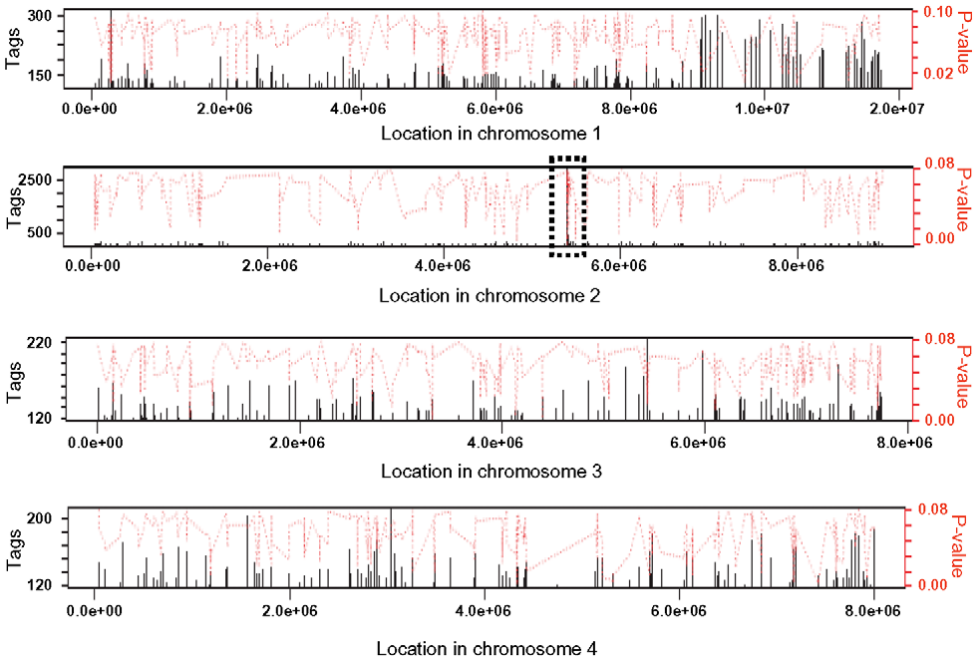
Browser shot of Tri6 binding sites in all four chromosome of *F. graminearum*. The binding sites were identified by the site identification software SISSR [22]. The black bars in each window refer to the number of tags or reads for a given site. The corresponding *P*-value is displayed in red. The dotted box in chromosome 2 indicates the site of the *Tri6* promoter region.

Tri6 has been defined as a pathway-specific transcription factor that regulates genes of the trichothecene gene cluster under nitrogen-deprived conditions. However, to characterize Tri6 as a global transcriptional regulator, we sought to identify targets of Tri6 by performing a genome wide ChIP-Seq in *F. graminearum* grown in nutrient-rich conditions. The ChIP-Seq was performed in the *Tri6-HA* complemented strain and was compared to the *Tri6*Δ strain. It should be noted that the addition of the HA epitope to the C-terminus of *Tri6* did not compromise its function [21]. Moreover, the presence of Tri6 protein in the *Tri6-HA* complemented strain was confirmed by immunoblot blot analysis using HA antibodies (Fig. S1A). ChIP DNA samples from the *Tri6-HA* and the *Tri6*Δ strains were sequenced by Illumina Genome Analyzer as 38 base tags. The software Novoalign was used to map the tags/reads to the reference genome (*F. graminearum*, PH-1; NRRL 31084) and the software Site Identification from Short Sequence Reads (SISSRs) was used to identify potential binding sites [22]. A browser shot of the output from the SISSRs analysis is displayed in Fig. 1. Among the 1491 enriched binding sites in the *Tri6-HA* complemented strain, we identified a total of 198 protein-coding genes with at least one binding site 1 Kb upstream of the ORF as potential targets of Tri6, distributed in all four chromosomes (Fig. 1). The binding site was defined by a high stringent criterion with a minimum of 120 tags and the number of tags per given target is proportional to the affinity of Tri6 to its target genes [22]. This is highlighted by the substantial enrichment of region in chromosome 2, where *Tri6* is located (dotted box, Fig. 1). The 198 target genes with their tags are listed in Table S1.

The analysis of the 198 target genes by the MIPS *F. graminearum* FunCat database (http://mips.helmholtzuenchen.de/genre/proj/DB/Search/Catalogs/searchCatfirstFun.html) and Kyoto encyclopaedia of genes and genomes (http://www.genome.jp/kegg/kegg1.html) categorized them into various aspects of metabolism and cellular processes (Table 1). For example, genes involved in nitrogen metabolism, such as pyridoxal decarboxylase (*FGSG_08249*) which decarboxylates L-glutamate into GABA and ornithine aminotransferase (*FGSG_02304*) which transaminates L-ornithine into glutamate- γ -semialdehyde were identified (Table S2) [23], [24]. Genes involved in lipid metabolism were also identified as potential targets of Tri6. For example, triacyl glycerol lipase (FGSG_02082) and acyl-CoA thioesterases (FGSG_03286 and FGSG_02848) yield free fatty acids, which are used in the β-oxidation pathway to produce energy [25]. In addition, acetyl-CoA, the by-product of thioesterase activity, is assimilated into the energy generating TCA cycle (Table S2) [25]. The data also revealed that Tri6 has the highest affinity towards its own promoter and to other genes of the trichothecene biosynthesis pathway, namely *Tri1, Tri14, Tri3, Tri12* and *Tri7* (Table 2). Since the *Tri* genes are normally induced in nutrient-limiting conditions, the discovery of these genes as targets of Tri6 in nutrient-rich conditions suggested a new role for *Tri6*.

**Table 1:** Tri6 targets summarized by functional categories.

Functional Category	Number of genes
01 Metabolism	25
02 Energy	3
10 Cell cycle and DNA processing	9
11 Transcription	9
12 Translation	3
14 Protein fate	12
16 Protein with binding function	23
18 Regulation of metabolism and protein function	3
20 Cellular transport, transport facilities and routes	24
20.01.10 Protein transport	4
20.01.27 Drug/toxin transport	5
20.03 Transport facilities	8
20.03.25 ABC transporters	3
20.09 Transport routes	13
30 Signal transduction	6
30.01.05.05 G-protein mediated	3
30.01.05.05.01 GTPase mediated	3
32 Cell rescue, defense and virulence	15
32.05 Disease, virulence and defense	7
32.05.03 Defense related proteins	4
32.07 Detoxification	6
34 Interaction with the environment	6
40 Cell fate	4
42 Biogenesis of cellular components	10
43 Cell type differentiation	6
99 Unclassified proteins	114

**Table 2:** Enrichment of Tri6 binding sites in the promoters of trichothecene genes in nutrient-rich conditions.

Tags	Trichothecene genes
88725	*FGSG_03536, Tri6*
6882	*FGSG_00071, Tri1*
5426	*FGSG_03543, Tri14*
362	*FGSG_03534, Tri3*
260	*FGSG_03541, Tri12*
206	*FGSG_03533, Tri7*

ChIP-Seq was performed with *Tri6-HA* complemented and the *tri6*Δstrains. The Tags represent binding of Tri6 protein to the promoters of *Tri* genes in the *Tri6-HA* strain in nutrient-rich conditions.

In addition to the structural genes involved in metabolism, the targets of Tri6 also included genes involved in regulatory functions and signal transduction processes (Table S2). Many of the transcription factors are classified as zinc-binding proteins and some are known to be involved in nitrogen regulation, including two genes (*FGSG_05942* and *FGSG_10774*) with NmrA domains. Genes with NmrA domains with Rossman fold structures can act as negative regulators of nitrogen catabolite repression [26], [27]. Two members of the RAS family of GTP binding proteins (*FGSG_01649* and *FGSG_06209*) and a homologue of *GIT1*, a member of the adenyl cyclase associated family of proteins (*FGSG_01923*), were identified as targets of Tri6. RAS has been shown previously to regulate growth and pathogenesis in *Fusarium* while *GIT1* in *S. pombe* is an essential component of the cAMP signalling pathway that primarily responds to glucose [28], [29].

In summary, the genome-wide ChIP-Seq performed in nutrient-rich conditions identified new targets involved in various aspects of metabolism. The targets encompassed not only regulatory genes, but also genes involved in primary and secondary metabolism, energy, and transport. The analysis also identified genes of the trichothecene gene cluster. This was particularly interesting given the fact that these genes are activated only in nutrient-deprived conditions. This suggested that that under nutrient-rich conditions, Tri6 could potentially exert transcriptional control over itself and other *Tri* genes.

### *Tri6* auto-regulates its own expression in nutrient-rich conditions

**Figure 2:**
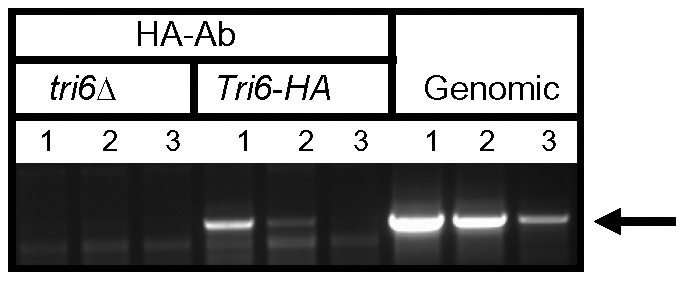
Tri6 binds to its cognate promoter *in vivo*. ChIP was performed with HA antibodies (HA-Ab) with the *Tri6-HA* complemented (*Tri6-HA*) and Tri6 mutant (*tri6*Δstrains. PCR was performed on the ChIP samples using the primers *FGSG_03536* Promo F and *FGSG_03536* Promo R, encompassing the 1.2 Kb upstream of the *Tri6* ORF, using different amounts of input DNA in the PCR reaction. Input DNA: Lane 1 = 25 ng, Lane 2  = 2.5 ng and Lane 3  = 0.25 ng of DNA. *Fusarium* genomic DNA (Genomic) was used as control to monitor the size of the PCR fragment (arrow). This is representative of two independent experiments.

The ChIP-Seq data suggested that Tri6 had high affinity to its own promoter so we were interested to know if Tri6 would bind to its own promoter and self-regulate its expression. To demonstrate that Tri6 protein binds to its own promoter, the DNA-Tri6 complex was immunoprecipitated with HA antibodies from both the *Tri6*Δ and the *Tri6-HA* complemented strains grown in nutrient-rich conditions and PCR was performed using the primers spanning the upstream region of *Tri6* ORF. As shown in Fig. 2, the primer set Tri6-Prom F/R (Table S3) amplified a product of 1.2 kb only from the samples immunoprecipitated from the *Tri6-HA* complemented strain (Lanes 1-3, *Tri6-HA*, Fig. 2). We could not amplify a 1.2 kb band in the samples immunoprecipitated from the *tri6*Δstrain (Lanes 1-3, *tri6*ΔFig. 2), even from 25 ng of input DNA (Lane 1, *tri6*ΔFig. 2). Genomic DNA was used as control to monitor the size of the PCR fragment (Lanes 1–3, Genomic, and Fig. 2). These *in vivo* results validated the Chip-Seq results and indicated that Tri6p can bind to its own promoter.

**Figure 3:**
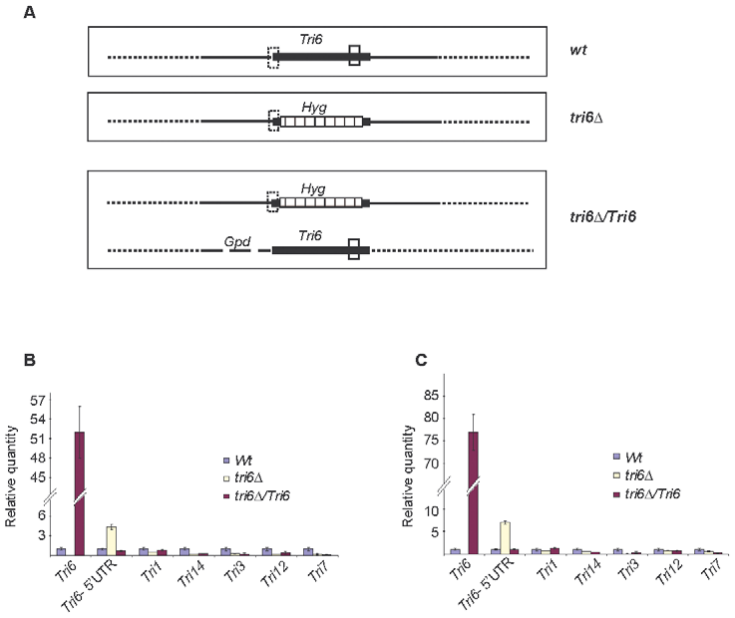
Tri6 auto-regulates its own expression in nutrient-rich conditions. **A**) Arrangement of the *Tri6* gene and location of Tri6 primers used in the RT-qPCR analysis of wildtype (*Wt*) and transgenic strains. The solid vertical box indicates the location of Tri6-ORF primers in the coding region of *Tri6* (Filled horizontal box) of the wildtype and the *Tri6* over-expressing transgenic strains (*tri6*Δ*Tri6*). The dotted vertical box indicates the location of Tri6 5′ UTR primers in the wildtype (*Wt*), the *Tri6* mutant (*tri6*Δand the *Tri6* over-expressing transgenic strains (*tri6*Δ*Tri6*). The location of the Hygromycin gene within the *Tri6* coding region of the *tri6*Δstrain (*tri6*Δis indicated by the striped horizontal box. Gpd indicates the promoter used to over-express*Tri6* [41]. The solid horizontal lines indicate 5′ and 3′ flanking regions of the *Tri6* gene. **B**) The quantitative real-time PCR (RT-qPCR) analysis of *Tri* genes in wildtype, *tri6*Δand the *tri6*Δ*Tri6* strains grown in nutrient-rich conditions. RT-qPCR reactions were performed in triplicates using Applied Biosystems Power SYBR Green kit and the Applied Biosystems Step One Plus Real-Time PCR System. A list of qPCR primers for all the *Tri* genes is listed in the Table S2. The β-tubulin gene (*FGSG_09530*) was used as the internal control and the data was imported and Relative quantity (RQ) was derived by the Relative standard method included in the StepOne 2.1 software. **C**) Identical to **B**), except the internal control used was *Gapdh* (*FGSG_06257*). The figures are representative of two independent biological replicates.

To demonstrate that Tri6 can bind to its own promoter and regulate its expression in nutrient-rich conditions, *Tri6* expression was monitored in the wildtype strain, the *tri6*Δstrain, and the strain that over expressed *Tri6* in the *tri6* mutant background strain (*tri6*Δ*Tri6)*. We designed two distinct primer sets (Table S3) to monitor *Tri6* transcripts. The first primer set (Tri6-ORF F/R) was designed in the coding region of *Tri6* (vertical open box, Fig. 3A) and as shown, over expression of *Tri6* in the *tri6*Δ strain (*tri6*Δ*Tri6)* led to a significant increase of *Tri6* transcripts compared to the wildtype strain (52±4, Tri6-ORF, Fig. 3B and 77±7; Tri6-ORF, Fig. 3C). As expected, no expression of *Tri6* was detected with these primers in the *tri6*Δstrain (Fig. 3B and 3C). The second primer set was designed to overlap the 5′UTR region and the coding region of *Tri6* (Dotted vertical box, Fig. 3A) which allowed us to monitor *Tri6* transcripts originating only in the wildtype and the *tri6*Δstrains. As shown in the Fig. 3B and Fig. 3C, a significant increase of *Tri6* expression (Tri6-5′UTR, 4.4±0.4, Fig. 3B and Tri6-5′UTR, 7±0.9, Fig. 3C) was observed in the *tri6*Δstrain, compared to the wildtype strain. However, over expression of *Tri6* in the *tri6*Δstrain (*tri6*Δ*Tri6)* resulted in decreased *Tri6* expression (Tri6-5′UTR, 0.72±0.1, Fig. 3B and Tri6-5′UTR, 1±0.06, Fig. 3C). This suggested that Tri6 acts as a repressor, regulating its own expression in nutrient-rich conditions. We did not observe any significant change in the expression of other *Tri* genes in the *tri6*Δ*Tri6* strain (*tri6*Δ*Tri6*; Fig. 3B and 3C), suggesting that additional factors are required for the expression of these genes.

### Identification and characterization of the Tri6 binding site in the *Tri6* promoter

**Figure 4:**
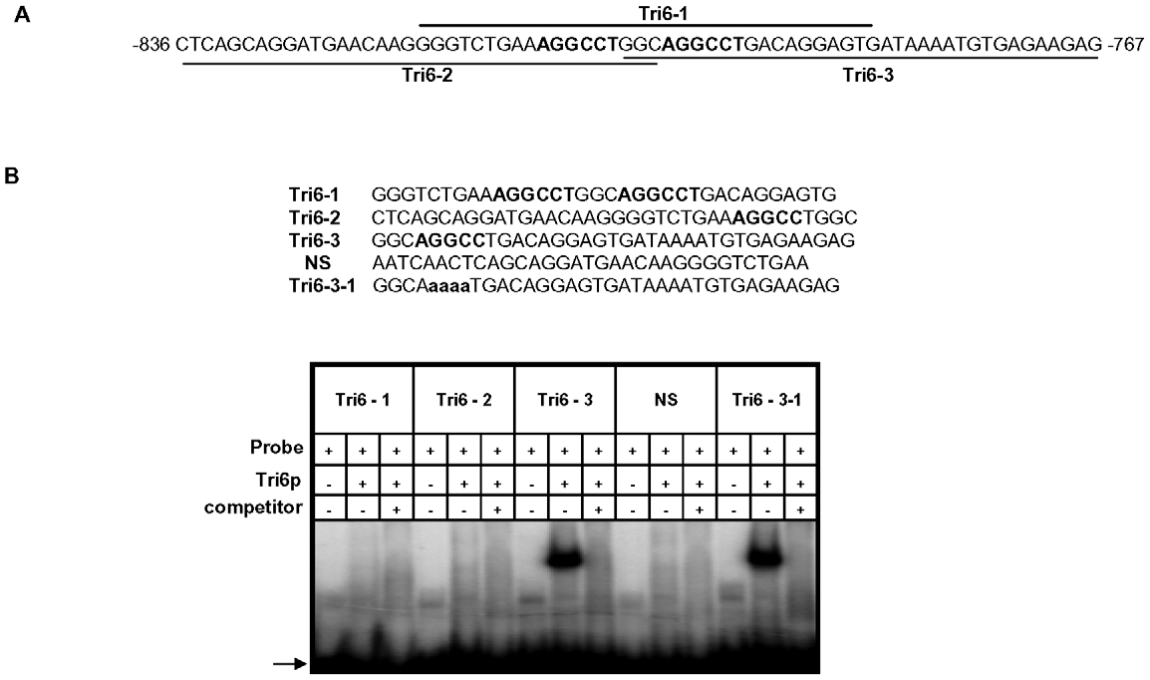
The Tri6 protein does not recognize the YNAGGCC motif by EMSA analyses. **A**) Sequence of probes Tri6-1, Tri6-2 and Tri6-3 in the *Tri6* promoter (-836 to -767) used in the EMSA analyses. The bold letters indicate the putative consensus binding sequence YNAGGCC for Tri6p. **B**) EMSA analyses were performed using the probes Tri6 -1, Tri6-2, Tri6-3, a non-specific probe (NS) and a probe with mutations within the binding motif (Tri6-3-1). The addition of Tri6 protein (Tri6p), the probe and the unlabelled probe (competitor) is indicated by ‘+’ sign and the omission is indicated by the ‘-’ sign. The capital bold letters indicate the consensus binding sequence and the bold non-capital letters represent mutations within consensus binding sequence YNAGGCC. Arrow indicates the migration of the free probe. This is representative of at least three independent experiments.

**Figure 5:**
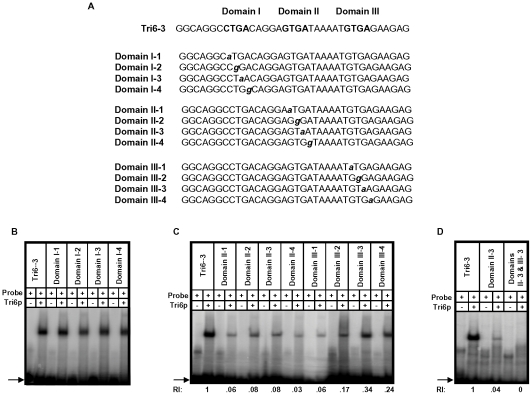
Tri6 binds to GTGA repeats in the *Tri6* promoter by EMSA analyses. **A**) Sequence of the probes used in the EMSA analyses. The three domains and the mutation within each Domain I, II, and III in the Tri6-3 probe are indicated. Bold capital letters indicate the three Domains in the Tri6-3 probe and the bold non-capital letters represent mutations in each Domain. **B**) Domain I with CTGA motif is not involved in Tri6p binding. EMSA analyses with individual mutation in the CTGA sequence was assessed for Tri6p binding. **C**) Mutations in the two GTGA domains represented by Domain II and Domain III, respectively, reduce Tri6p binding. EMSA analyses with individual mutation in the GTGA sequence in both domains were assessed for Tri6p binding. **D**) Domain II and III are required for complete Tri6 binding. A combination of mutations in Domain II and III (Domain II-3 and Domain III-3) were assessed for Tri6 binding. Tri6-3 was used as positive control and Domain II-3 was used as reference. The addition of Tri6 protein (Tri6p) and the probe is indicated by ‘+’ sign and the omission is indicated by the ‘-’ sign. RI represents relative intensity and is the densitometer reading corresponding to the binding of Tri6p to each probe with respect to the control probe Tri6-3. The arrow represents the migration of free probe. This is representative of at least three independent experiments.

A previous study performed in *F. sporotrichioides* suggested TNAGGCC as a DNA binding site for Tri6 protein [17]. A recent study that examined the promoters of genes differentially regulated by *Tri6* during the *F. graminearum* infection process also suggested a similar DNA binding motif [16]. The results described here confirmed that Tri6 is able to bind its own promoter *in vivo* (Fig. 2) and regulate its own expression (Fig. 3). Since the promoter of *Tri6* (−836 to −768) harbours two RNAGGCC (where R =  G or A) binding sites (Fig. 4A), we employed EMSA analyses to further delineate the binding site for Tri6. All the EMSA assays were performed with purified recombinant Tri6 protein (Fig. S1B). First, we tested the probe which contained two of the RNAGGCC motifs and as the results indicated, Tri6 did not bind to this probe (Tri6-1, Fig. 4B). This prompted us to examine the region that surrounds this motif. The probe Tri6-2 which included sequences proximal to the binding sites (Fig. 4A) also did not bind the Tri6p (Fig. 4B). However, a probe (Tri6-3) which included sequences distal to the binding sites was able to bind Tri6 protein. The specificity of the binding to the Tri6-3 probe was confirmed by the addition of 10-fold excess non-labelled Tri6-3 probe in the EMSA assays (Tri6-3, competitor, ‘+’, Fig. 4B). In addition, we also used a probe which did not contain this consensus sequence as a negative control (Tri6-NS, Fig. 4B). These results suggested that RNAGGCC motif is not involved in Tri6 binding *in vitro*. To further confirm this, nucleotides GGCC within the motif were mutated in the Tri6-3 probe and the results indicated that the mutations did not abolish Tri6 binding (Tri6-3-1, Fig. 2). These results confirmed that the YNAGGCC motif is not required for binding of Tri6, but suggested that nucleotides outside of this motif in the Tri6-3 probe are involved in Tri6 binding. Outside of the RNAGGCC motif, three domains in the Tri6-3 probe were recognized that could potentially bind Tri6 (Fig. 5A). We designated CTGA sequence which partially overlaps the AGGCC site as Domain I and the two GTGA repeats separated by six nucleotides as Domain II and III, respectively (Fig. 5A). As the EMSA results indicated, individual nucleotide mutations within Domain I did not have any noticeable effect on Tri6 binding (Fig. 5B). However, individual mutations in Domain II and Domain III of the GTGA sequences, respectively, dramatically decreased Tri6 binding (Fig. 5C). Furthermore, combined mutations in both Domain II and III completely abolished the Tri6 binding (Fig. 5D). These results suggested that either a single GTGA sequence or GTGA repeats in the promoter of *Tri6* are required for Tri6 binding.

### Tri6 targets are differentially regulated under nitrogen limiting conditions

**Figure 6:**
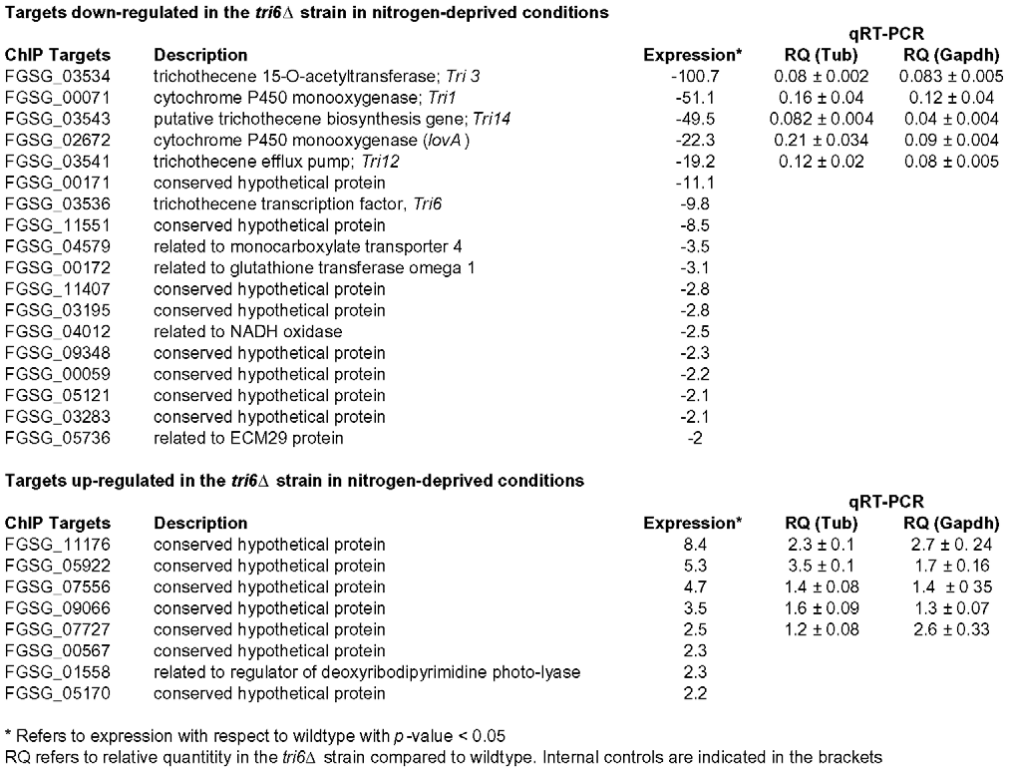
*Tri6* differentially regulates 26 targets under nitrogen-deprived conditions. Expression profiling was performed with wildtype and *Tri6* mutant (*tri6*Δstrain grown in nitrogen-limiting conditions. Eighteen targets that are down-regulated and eight targets that are up-regulated in the *tri6*Δstrain are represented. The top five genes in each category were verified by RT-qPCR analysis represented by relative quantity (RQ). Two internal standards (*β- tubulin* and *Gapdh*) were used. This is a representative of three independent experiments.

The 198 Tri6 targets identified in nutrient-rich conditions included several genes of the trichothecene gene cluster (Table 2). Since the *Tri* genes are expressed only under nitrogen-limiting conditions, we were interested to know how many of the non-*Tri* gene targets are co-regulated with the *Tri* genes. To address this, we performed a genome-wide expression analysis of wildtype and *tri6*Δstrains grown in nitrogen-limiting conditions. As the microarray analysis indicated, we identified a total of 1614 genes that were differentially regulated in the *tri6*Δstrain (2-fold cut off; Table S4). Among the 870 down-regulated genes, 18 of the *Tri6* targets were represented and another eight were represented in the 744 up-regulated genes (Fig. 6). Top five genes from each of the up and -down-regulated genes from the microarray analyses were selected for validation by RT-qPCR analyses (Fig. 6). If the expression threshold was set at 1.5, 49 of the 198 target genes (∼25% of the ChIP targets) were shown to be differentially regulated by *Tri6* in this nutrient-limiting condition (Table S5). Out of 49 targets, 23 genes or ∼53% were annotated as unclassified by the MIPS functional annotation program and although others were classified into four major groups, only one category associated with cellular transport comprised of eight target genes were enriched to a significant level (enrichment of 19% vs 10% in the genome, *p*-value 0.06). Thus expression profiling provided a strong evidence to suggest that *Tri6* extends its regulatory control beyond the trichothecene cluster and additionally, Tri6 can act both as a positive and negative regulator.

## Discussion

Much of our understanding regarding the transcription regulator Tri6 stems from earlier studies that identified its control of the mycotoxin pathway leading to trichothecene biosynthesis and designated it as a pathway-specific transcriptional regulator [17], [18]. Since then, evidence from numerous laboratories have challenged this perception and thus prompted us to hypothesize that Tri6 is a global transcriptional regulator whose influence extends beyond the trichothecene gene cluster [16], [20]. Accordingly, the ChIP-Seq experiments performed in nutrient-rich conditions demonstrated that Tri6 could bind to promoters of both structural and regulatory genes associated with many aspects of nitrogen, carbon and lipid metabolisms (Table S2). One of the intriguing revelations was that even under these conditions Tri6 was bound to the promoters of genes involved in the biosynthesis of the secondary metabolite, trichothecene 15-ADON (Table 2). Since this cluster is only activated in *Fusarium* grown under nitrogen-deficient conditions, binding of Tri6 to the promoters of *Tri* genes suggested that Tri6 could potentially act as a negative regulator, suppressing these genes under nutrient-rich conditions. The results from the RT-qPCR clearly demonstrated that with the exception of *Tri6*, none of the other *Tri* genes are subject to negative regulation by *Tri6* (Fig. 3). Since the switch from nutrient-rich to deprived conditions led to differential regulation of the target genes, we conclude that Tri6 can act both as a positive and a negative regulator. Furthermore, since the regulation by Tri6 extended beyond the trichothecene gene cluster, we propose that Tri6 is a global transcriptional regulator.

### Tri6 and other global transcriptional regulators

Nitrogen catabolite repression, or NCR, refers to genes that are repressed when a preferred source of nitrogen such as ammonia or glutamine are present in the environment [24]. NCR is mediated by the action of global nitrogen regulators which regulate the expression of structural genes required to metabolize alternate nitrogen sources. *AreA* is a prototypic NCR gene regulator and has been identified in many fungi, including *A*. *nidulans*, homologues *Nut1* from *M*. *grisea* and *AreA-GF* from *F. fujikuroi* [30]–[33]. Under nutrient-rich conditions, expression of *AreA* is repressed by the binding of the repressor protein NmrA to the promoter of *AreA* [31], [32]. However, under nitrogen-starving conditions, *AreA* repression is relieved and target genes involved in the utilization of non-preferred sources of nitrogen are expressed [31], [32]. Coincidentally, some of the known *AreA* target genes such as the branched amino acid transferase (*FGSG_05696*; ∼2.2 –fold, Table S4) and the general amino acid permease (*FGSG_05574*; ∼4-fold, Table S4) that are normally expressed in the wildtype *Fusarium* strain under nitrogen-deprived conditions are repressed in the *tri6*Δstrain [34]. This leads one to speculate that *Tri6*, similar to *AreA*, is involved in NCR. Studies in *F. fujikuroi* have also shown that *AreA*, in addition to regulating those genes involved in NCR, also regulates expression of genes that synthesize secondary metabolites like gibberellins and the pigment bikaverin [35], [36]. An additional feature that is shared between *Tri6* and *AreA* is that they are both subject to auto-regulation [31]. Taken together, our evidence suggests one possible scenario where *Tri6* regulates functions of *AreA* through *NmrA* in *F. graminearum*. In support, we identified two genes (*FGSG_05942* and *FGSG_10774*, Table S2) with Rossman-fold domains as targets of Tri6. Rossman-folds are distinguishing features of NmrA proteins [26]. A further link between *NmrA* and virulence was recently established in *F. oxysporum* where the deletion of the transcription factor *MeaB*, which regulates *Nmr1*, an orthologue of *NmrA* resulted in the repression of the virulence gene *Six1* [37].

### DNA binding properties of Tri6

**Figure 7:**
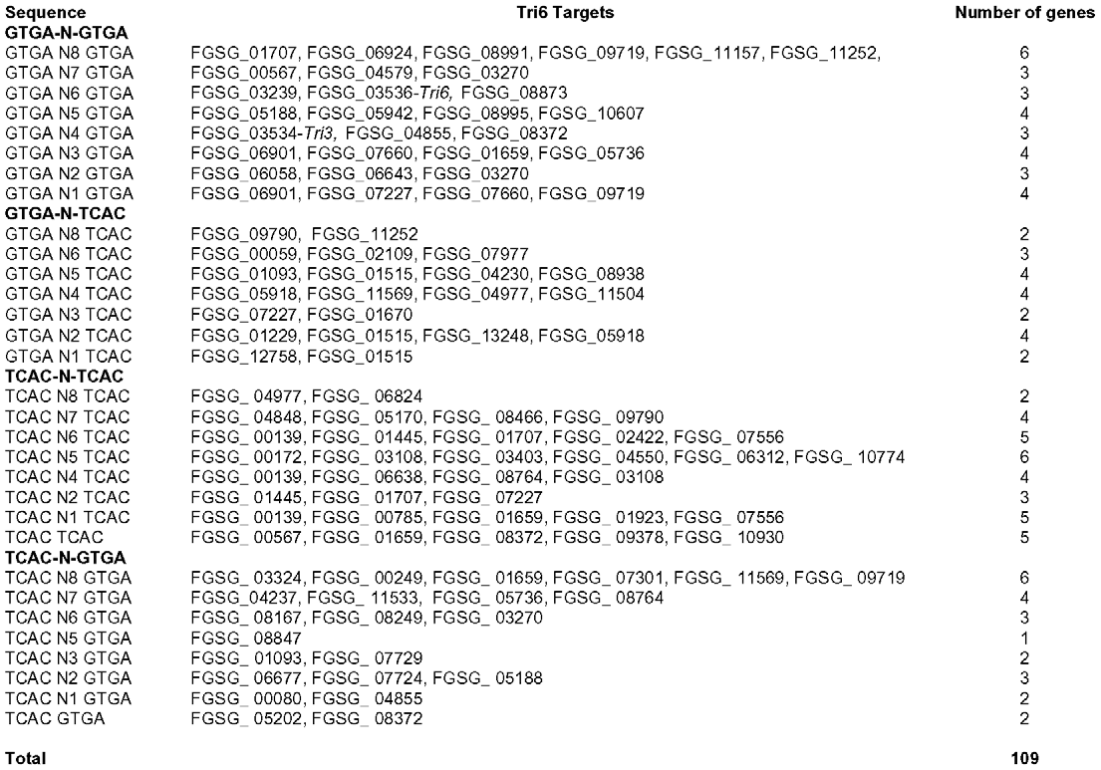
GTGA/TCAC repeats are present in the promoters of Tri6 target genes. One kilobase region upstream of the open reading frame of each of the Tri6 target genes were analyzed for GTGA/TCAC repeats separated by 0 to 8 nucleotides (N).

The EMSA studies demonstrated that mutations in either one of the GTGA elements in the promoter of *Tri6* reduced the binding of Tri6. However, combined mutations in both the GTGA elements completely abolished this binding (Fig. 5D). This suggested that the presence of two GTGA elements in close proximity to each other in the *Tri6* promoter likely contributes to the affinity of Tri6 to this site. Studies with other global regulators, specifically those responding to nitrogen, such as AreA in *A. nidulans* or its counterpart Nit2 in *N. crassa* showed that binding sites located within 30bp of each other in either orientation enhances their DNA binding affinity [38]. When these criteria were applied to the analysis of Tri6 target genes, 109 of the 198 target genes harboured multiple GTGA/TCAC binding sites in their promoters, separated by eight nucleotides or less, suggesting a common mechanism of binding and regulation by *Tri6* (Fig. 7). It should be noted that GTGA/TCAC motifs separated by more than eight nucleotides are represented in the promoters of all the Tri6 target genes (Table S1). The GTGA/TCAC motif described in this study is distinct from the previously reported YNAGGCC for Tri6 binding [17]. Unlike this study, Hohn et al. did not use purified Tri6 protein and furthermore, their EMSA analysis with regions of the *Tri3* promoter which contained the putative YNAGGCC consensus binding site did not result in a shift [17]. This led them to speculate an alternate binding site for Tri6. It is noteworthy to indicate that *Tri3* is one of the genes identified as targets of Tri6 with the GTGA repeats in its promoter.

Studies with global regulators have outlined several mechanisms that influence both binding affinity and specificity [31]. For example, proteins that binds to direct repeats most likely bind as dimers. As an example, Dal80 protein in *S. cerevisiae*, involved in nitrogen catabolism, binds to two closely spaced GATA elements as a dimer in either a tail-to-tail or head-to-head orientation [39]. Interactions between global and pathway-specific regulators also influence both DNA binding specificity and affinity. For example, some of the genes involved in nitrate assimilation are regulated by the interaction between the global regulators AreA and NirA in *A. nidulans*, while other genes are regulated by the global regulator Nit2 and the pathway-specific regulator Nit4 in *N. crassa* [24]. Since we observed closely spaced GTGA repeats in the *Tri6* promoter of *F*. *graminearum*, we could envisage a scenario, where Tri6 could function as a dimer and repress the expression of *Tri6* in nutrient-rich conditions. However, under nutrient-deprived conditions, where the expression of *Tri6* increases, Tri6 could interact with other known regulators such as Tri10 or PacC to provide specificity and regulate expression in condition-specific manner [14], [19]. This interaction may also provide a platform for the Tri6-complexes to bind to an alternate site, such as the YNAGGCC proposed in several studies. Such a scenario could account for the differences between the genes regulated by *Tri6* in this study from those seen in a recent study of *F. graminearum* infection *in planta* [16]. It is noteworthy to mention that the authors in that study also observed stark differences between the genes regulated by *Tri6* in culture and *in planta*, albeit the comparison was made between two different *Fusarium* strains. One of the intriguing findings from the *in planta* study was that the promoters of genes, specifically the genes of the isoprenoid pathway essential for trichothecene production in both *in planta* and culture, showed enrichment of the YNAGGCC motif only in *F. graminearum* and not in other *Fusarium* species unable to make trichothecenes or in other related fungi [16]. It was proposed that co-regulation of the isoprenoid genes and the trichothecene genes represent an evolutionary adaptation specific to *F. graminearum*. A logical extension of this argument is that the source of nutrition, determined by the environment such as culture conditions or the type of host that this fungus infects, is the driving force behind this adaptation.

In conclusion, this study has identified new or additional role for the transcriptional regulator, Tri6. The findings from this study together with earlier studies have led us to conceive a new mode of action for Tri6. In this model, Tri6 recognizes and binds the GTGA/TCAC elements under one condition such as the nutrient-rich condition where it regulates itself and other genes involved in various aspects of metabolism. However, when the environmental conditions change, be it in culture or *in planta*, activation of other regulators including Tri10 and PacC may change the dynamics of Tri6p-complex and initiate new interactions with the DNA, which could involve recognition of an alternate site such as the YNAGGCC motif. Although, we have no evidence to show that Tri6 directly interacts with either Tri10 or PacC, isolation and identification of Tri6 and Tri10 protein complexes *in vivo* will shed more light on both the dynamics and the links between these regulators.

## Materials and Methods

### Strains and growing conditions

*Fusarium graminearum* wildtype strain GZ3639 (NRRL 38155) was provided by C. Babcock of the Canadian Collection of Fungal Cultures (CCFC), Agriculture and Agri-Food Canada, Ottawa. *F. graminearum* and transformants were grown on PDA (Sigma Chemical Co. USA) plates.

### Construction of *Tri6*Δdeletion, *Tri6-HA* complement and *tri6*Δ/*Tri6* over expression strains

Constructs for the *tri6*Δand *Tri6-HA* complement strains have been described (Fig. S1; http://apsjournals.apsnet.org/doi/suppl/10.1094/MPMI-09-10-0210). To construct the Tri6 over expression vector, we first PCR amplified *Tri6 ORF* with the primer set Tri6-GUE-F/ Tri6-GUE-R (Table S2) and *F. graminearum* genomic DNA as template. The PCR fragment was cloned into the vector pSW-GU. The backbone of pSW-GU vector is identical to pRF-HUE [40] except that the selection marker Hygromycin gene was replaced with Geneticin, encoded by the gene neomycin phosphotransferase [40]. Transformation of the *tri6*Δstrain with the *Tri6* over expression vector was performed according to Rasmus et al [40]. The transgenic *Fusarium* strains were verified by PCR (Fig. S2). PCR conditions: 200 µM Primers, 200 µM NTP's, 1 unit of Expand long Taq polymerase (Roche), 95°C for 30 s, 52°C for 30 s for annealing, 68°C for extension for 37 cycles. All PCR products were purified using Qiagen's PCR purification kit.

### Trichothecene induction and detection

To induce trichothecene production in liquid culture, a two stage media protocol, modified from Miller and Blackwell was employed [41]. 20,000 spores of wildtype, *tri6*Δand *tri6*Δ*Tri6* over expression strains were inoculated into 4 mL of first stage growth media and incubated in Falcon Multiwell 6-well culture trays (Fig. S2). The culture trays were affixed to an orbital shaker and the spores were grown for 24 hr at 28°C in the dark with constant shaking at 170 rpm. Following 24 hr growth, the mycelial solids were washed with water and resuspended in 4 mL of second stage media (pH 4.0) [41] and then transferred to the 6-well culture trays. The mycelium was grown in second stage media under the same conditions as described previously. The supernatant was collected after 24 hr for trichothecene analyses. Trichothecenes were analysed on an AKTA 10 purifier (GE Healthcare, Canada) with direct injection of 100 µL of the culture filtrate into a 150×4.6 mm, 5 µm Hypersil ODS column (Thermo-Electron Corp.), using a methanol: water gradient from 15∶85 to 60∶40 over 25 min at a flow rate of 1 ml/min. Under these conditions, 15-ADON elutes at a retention time ∼10 min, monitored by UV 220_nm_.

### Chromatin Immunoprecipitation

Spores from the *tri6*Δand the complemented (*Tri6-HA*) strains were used to inoculate first stage media and grown for 19 hr as described before. The mycelia were washed with water and filtered (sterile 1MM; Whatman). The ChIP-enriched DNA was prepared according to Saleh et al. [42] with modifications. The mycelial pellet was incubated in the cross-linking buffer for 30 min with continuous shaking. After thorough washing, the pellet was ground in liquid N_2_ and resuspended in the lysis buffer (250 mM, HEPES pH 7.5, 150 mM NaCl, 1 mM EDTA, 1% Triton, 0.1% DeoxyCholate, 10 mM DTT and Protease inhibitor (Roche complete Mini) and incubated for 1 hr on ice. To obtain uniform fragments of cross-linked DNA, the suspension was sonicated 6x for 15 sec (60% amplitude, Misonix 4500 Sonicator). A small aliquot was used to confirm a dominant diffused band between 500 and 1000 bp. Approximately 750 µg of protein was incubated with 50 µL HA-magnetic beads (Miltenyi Biotech) and left overnight at 4°C. Immunoprecipitates were washed six times with lysis buffer and eluted with 250 µL of freshly prepared elution buffer (0.5% SDS and 0.1 M NaHCO_3_) with incubation at room temperature (RT) for 15 min with gentle agitation. The elution step was repeated once with 30 min of incubation at RT. To reverse the cross-linking, 20 µL of 5 M NaCl was added to the 500 µL elutes and incubated overnight at 65°C. The samples were further incubated for 1.5 hr at 45°C with 1 µL of Proteinase K (20 mg/mL), 30 mM Tris-HCl pH 6.5 and 10 mM EDTA to digest the proteins. DNA from these samples was precipitated according to Saleh et al. [42].

### Preparation of DGE and ChIP libraries and Illumina sequencing

ChIP- enriched DNA was prepared for sequencing using the Illumina ChIP sample prep protocol with minor modifications. In brief, 2 µg of ChIP-enriched DNA was end repaired, and an adenosine overhang added to the 3′ ends. Standard Illumina adapters were ligated to the DNA and 240 bp fragments size selected from a 1.5% agarose gel. The eluted products were enriched using 18 cycles of amplification with Illumina PCR primers. Libraries were validated on the Bioanalyzer 2100 (Agilent) and Qubit fluorometer (Invitrogen, Carlsbad, CA). Each sample was run on two lanes of the Illumina GAII sequencer at the Centre for the Analysis of Genome Evolution and Function (CAGEF, University of Toronto). For each sample, 6 pmol was loaded in one lane of a standard flow cell, and 8 pmol was loaded in a second lane. The GAII was run for 38 cycles.

### Statistical analysis of ChIP data

In total, 24,201,991 out of 29,933,903 tags from *Tri6-HA* complemented strain and 15,756,703 out of 22,010,411 tags from *tri6*Δstrain were normalized and uniquely mapped to the genome by Novoalign (http://www.novocraft.com) [43], [44]. The rest of the tags were either of low quality or mapped to multiple locations. Of the mapped tags, ∼50% were mapped to sense strand and another 50% were mapped to anti-sense strand. The mapping data were analyzed by the Site Identification from Short Sequence Reads (SISSRs) software for identification of the binding sites with tags originating from the *tri6*Δstrain as control [22]. The following parameters were used to run the program: 36,898,000 base pairs for the genome size, 0.8 for the fraction of genome mappable by reads, 1 for the E-value (minimum number of directional tags required on each side of the inferred binding site), 0.1 for the p-value (for fold enrichment of ChIP tags at a binding site location compared to that at the same location in the control data), 2 for the scanning window size, 240 base pairs for the average DNA fragment length, and 350 base pairs for the upper bound DNA fragment length. The switch -u was turned on to allow for identification of the binding sites supported only by reads mapped to one strand. Under these conditions, we were able to identify 1491 potential binding sites with at least 120 tags (Tag#, the sum of the tags mapped to positive strand of the left half of the binding site and the tags mapped to negative strand of the right half of the binding site) and at least 2.15 fold more tags in treatment vs control. We then compiled a list of 198 protein-coding genes with at least one binding site in 1 Kb upstream of the start site. The sum of the Tag# indicates the relative affinity of Tri6 to the binding sites in the promoter of the gene.

### RNA isolation and Quantitative RT-PCR Analysis

The two-stage media was employed to grow *F. graminearum* as described before. Mycelia grown for 5 hr in the second stage (nitrogen-limiting) media were filtered and ground to a powder in liquid nitrogen. The RNA was isolated from 0.25 gm of ground mycelia with 1 mL of Trizol (Invitrogen, USA) according to manufacturer's instructions. The RNA was purified further with the InviTrap Spin Cell RNA mini kit (Invitek, Germany) according to the manufacture's instructions. cDNA for the quantitative RT-PCR Analysis (RT-qPCR) experiments was synthesized from 1 µg total RNA using random hexamers using the high-capacity cDNA reverse transcription kit (Applied Biosystems, USA). RT-qPCR was performed using the Applied Biosystems StepOne Plus Real Time PCR system (ABI, Foster City, USA). Standard curves were created for the house keeping genes (*β-tubulin; FGSG_09530 and Gapdh; FGSG_06257*) and the genes of interest according to Relative standard curve method outlined in StepOne 2.1 software. Primers used in the RT-qPCR are listed in supplementary Table S2. Using the relative standard curve, the relative quantity (RQ) was determined by comparing target quantity in each sample to the reference sample. P values <0.05 were considered to be statistically significant.

### Purification of Tri6 protein and Electromobility shift assays

*Tri6* was cloned into pDEST17 vector (Invitrogen, USA) and expressed in BL21 pLys *E*.*coli* and the Tri6 protein was purified by the His Trap FF affinity column (GE Health care, Sweden). Double stranded DNA probes (100 ηg/mL) were labelled using T4 Polynucleotide Kinase (Fermentas MBI) and 20 µci of γP^32^ (Perkin Elmer, USA). The labelled probes were purified using the Quick spin column (Roche, USA) and specific activity varied from 4–7×10^4^ cpm/ηg. Binding reactions for the EMSA were performed in 15 µL volume with 2 µg purified Tri6-HA recombinant protein, labelled probes (20,000 cpm; 2ηg) for 30 minutes at room temperature in a buffer containing 20 mM HEPES pH 7.9, 2 mM DTT, 5% (V/V) Glycerol and 1 µg poly(dIdT). The reaction was run on 6% polyacrylamide gels (acrylamide/bis ratio of 19∶1 w/w) in 50 mM Tris-HCl, pH 8.0, 50 mM sodium borate and 1 mM EDTA. The competition experiments were performed by adding unlabelled oligonucleotides to the reaction at 10X molar excess (20 ηg) of the labelled probe. The shift obtained by the Tri6 was scanned by the Storm 840 densitometer (GE healthcare, USA) and quantified by Image Quant TL 7.0 software (GE healthcare, USA). All the DNA probes used in EMSA were synthesized by Sigma Genosys (SigmaAldrich, Canada).

### DNA microarray and statistical analysis

Wildtype and the *tri6*Δstrains were grown in the second stage media for 5 hrs and the RNA was extracted as described before. The integrity of RNA was confirmed by an Agilent 2100 Bioanalyzer (Agilent, Canada). RNA was then converted into cDNA using the Agilent Quick Amp Labeling Kit and converted back into labeled RNA using T7 RNA polymerase and cyanine 3-labeled CTP or 5-lableled CTP from the Agilent two-color RNA Spike-in kit. The cRNA was then hybridized to a custom *F. graminearum* 4X44K oligomer microarray (Agilent Technologies, CA, USA), using an Agilent gene expression hybridization kit. Both dye combinations were performed for each biological replicate. Each array consisted of 1,417 spike-in and negative controls and up to three 60-mer oligos designed for each of 13,918 predicted *F. graminearum* genes (NCBI GEO, Platform Accession #GPL11046). The hybridizations were scanned using the GenePix Professional 4200A scanner, and the signals quantified using GenePix Pro 6. The microarray data was transferred into Acuity 4.0 and the data was normalized using Lowess Normalization. Data points with low intensities were removed, and the dye swapped replicates were combined and expressed in log_2_ ratio_._ ANOVA was used to determine the consistency between samples for each hybridization (P<0.05). The data points were then averaged first between three biological replicates, then within the three average hybridizations. The data was then back transformed from the log scale as fold-expression. Separate datasets were then generated that contained genes that were positively identified in the chromatin immunoprecipitation data, as well as the genes that are up and down regulated in the entire genome by either 1.5-fold or greater and 2.0-fold or greater. Raw data can be accessed at NCBI GEO, Accession # GSE30892.

## Supporting Information

Figure S1 **A)** **Detection of Tri6 protein in the Tri6 complemented strains (** ***Tri6-HA*** **) by immunoblot analysis.** 100 µg of total protein from both *Tri6* mutant (*tri6*Δand *Tri6* complemented strains (*Tri6-HA*) was separated by SDS-PAGE and detected by HA-antibodies. The arrow indicates the migration of the Tri6 protein and the * indicates non-specific cross reacting with HA antibodies and serves as internal loading control. **B)** **Purification of Tri6 protein expressed in bacteria.** *Tri6* gene was His tagged at the C-terminus and expressed in BL21-pLys *E. coli* and purified over a Nickel affinity column. Tri6 protein eluted from the Nickel affinity column was detected by Coommassie Blue G-250.
(TIF)

Figure S2 **A) Characterization of** ***Tri6*** **over expression strains by PCR.** Genomic DNA was isolated from wildtype (*Wt*), *Tri6* mutant (*tri6*strains and the *Tri6* over expresssor transgenic strain (*tri6*Δ*Tri6*). PCR was performed as outlined in the methods section with the primer set Tri6-ORF-F/Tri6-ORF-R to detect *Tri6* (lane 1), the primer set HygF/HygR to detect the selection marker Hygromycin (lane 2), the primer set GenF/GenR to detect the selection marker Geneticin (lane 3) and the primer set Tri6 GUE F/Tri6 GUE R to detect the entire *Tri6* over expression construct (lane 4). **B)** **HPLC analysis of the production of 15-ADON from all three strains grown in DON-inducing media**. The strains grown in six well culture plates and induced for 15-ADON as described in Methods. The retention time for the elution 15-ADON was ∼10 mins and the elution from each strain is indicated. There is no production of 15-ADON from the *tri6*Δstrain (Blue line). The quantity of 15-ADON is indicated by arbitrary absorbance units measured at 220 ηm (AU_220_).
(TIF)

Table S1 **Complete list of Tri6 targets identified in ChIP-Seq experiment.** The Tri6 gene targets are presented with gene description and the number of associated tags in the ChIP experiment. Number of tags is proportional to the binding affinity of Tri6 to their cognate promoter DNA sequences. * refers to the number of GTGA and TCAC sequence present in the one kb promoter of each gene.
(XLS)

Table S2 **Tri6 targets organized by functional category as determined by MIPS.**
(XLS)

Table S3 **List of primers used in this study.**
(XLS)

Table S4 **Complete list of** ***Fusarium*** **genes differentially expressed under nitrogen-deprived conditions.** Genes expressed in the *tri6*Δstrain relative to expression in the wildtype *Fusarium* strain and values represent expression over 2-fold or higher.
(XLS)

Table S5 **Differential expression of** ***Tri6*** **targets with cut-off of 1.5- fold or higher.**
(XLS)
