# Supplementary figures and images for: Tri6 Is a Global Transcription Regulator in the Phytopathogen Fusarium graminearum
Source: PLoS Pathog. 2011 Sep 29;7(9):e1002266. doi: 10.1371/journal.ppat.1002266 (PMC3182926; doi:10.1371/journal.ppat.1002266)

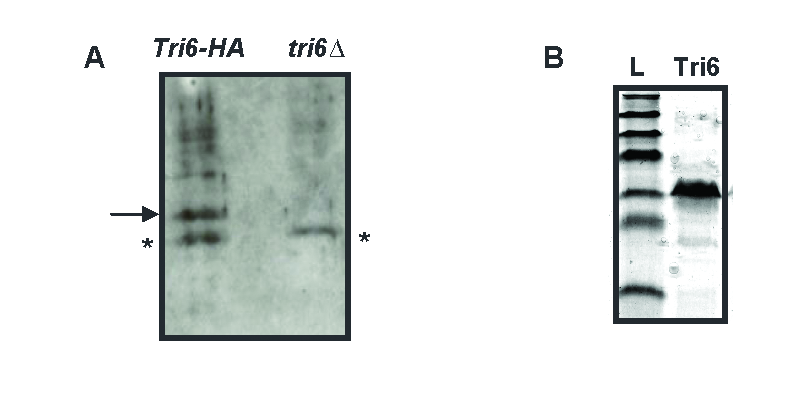

Supplement: Figure S1 — A) Detection of Tri6 protein in the Tri6 complemented strains ( Tri6-HA ) by immunoblot analysis. 100 µg of total protein from both Tri6 mutant (tri6Δand Tri6 complemented strains (Tri6-HA) was separated by SDS-PAGE and detected by HA-antibodies. The arrow indicates the migration of the Tri6 protein and the * indicates non-specific cross reacting with HA antibodies and serves as internal loading control. B) Purification of Tri6 protein expressed in bacteria. Tri6 gene was His tagged at the C-terminus and expressed in BL21-pLys E. coli and purified over a Nickel affinity column. Tri6 protein eluted from the Nickel affinity column was detected by Coommassie Blue G-250. (TIF) [file ppat.1002266.s001.tif]

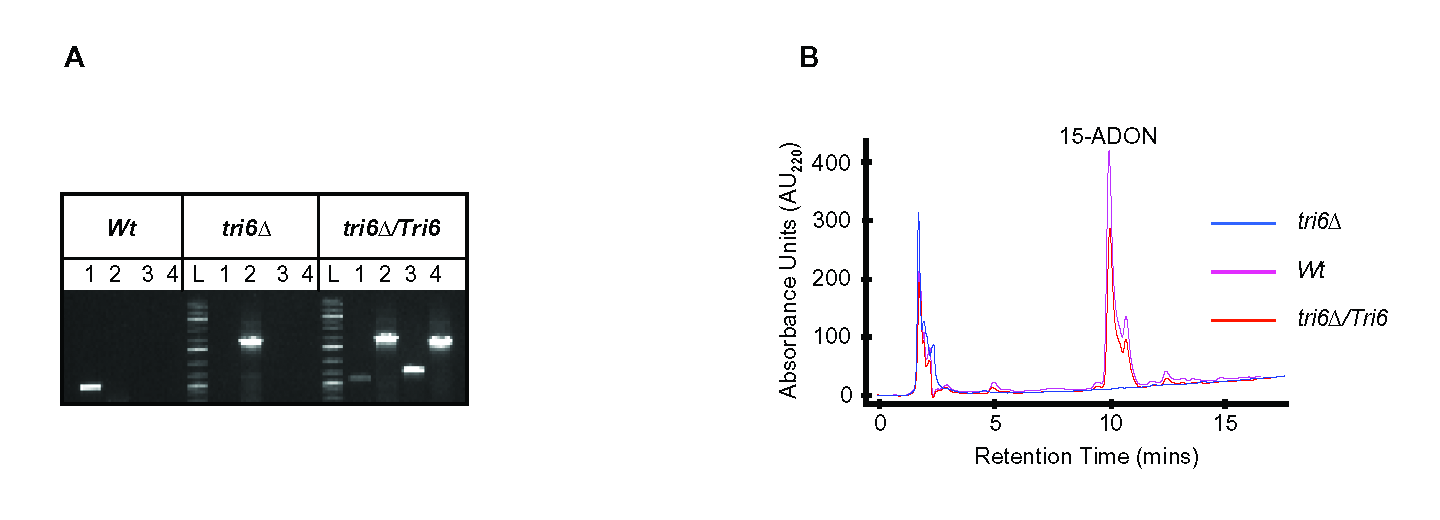

Supplement: Figure S2 — A) Characterization of Tri6 over expression strains by PCR. Genomic DNA was isolated from wildtype (Wt), Tri6 mutant (tri6strains and the Tri6 over expresssor transgenic strain (tri6ΔTri6). PCR was performed as outlined in the methods section with the primer set Tri6-ORF-F/Tri6-ORF-R to detect Tri6 (lane 1), the primer set HygF/HygR to detect the selection marker Hygromycin (lane 2), the primer set GenF/GenR to detect the selection marker Geneticin (lane 3) and the primer set Tri6 GUE F/Tri6 GUE R to detect the entire Tri6 over expression construct (lane 4). B) HPLC analysis of the production of 15-ADON from all three strains grown in DON-inducing media. The strains grown in six well culture plates and induced for 15-ADON as described in Methods. The retention time for the elution 15-ADON was ∼10 mins and the elution from each strain is indicated. There is no production of 15-ADON from the tri6Δstrain (Blue line). The quantity of 15-ADON is indicated by arbitrary absorbance units measured at 220 ηm (AU220). (TIF) [file ppat.1002266.s002.tif]
